# Early primary care physician contact and health service utilisation in a large sample of recently released ex-prisoners in Australia: prospective cohort study

**DOI:** 10.1136/bmjopen-2015-008021

**Published:** 2015-06-11

**Authors:** Jesse T Young, Diane Arnold-Reed, David Preen, Max Bulsara, Nick Lennox, Stuart A Kinner

**Affiliations:** 1 Centre for Health Services Research, School of Population Health, The University of Western Australia, Perth, Western Australia, Australia; 2 Melbourne School of Population and Global Health, The University of Melbourne, Carlton, Victoria, Australia; 3 National Drug Research Institute, Curtin University, Perth, Western Australia, Australia; 4 General Practice and Primary Health Care Research, School of Medicine, The University of Notre Dame Australia, Fremantle, Western Australia, Australia; 5 Institute for Health Research, The University of Notre Dame Australia, Fremantle, Western Australia, Australia; 6 Queensland Centre for Intellectual and Developmental Disability, School of Medicine, The University of Queensland, Mater Misericordiae Hospital, South Brisbane, Queensland, Australia; 7 School of Medicine, the University of Queensland, Herston, Queensland, Australia; 8 School of Public Health and Preventive Medicine, Monash University, The Alfred Centre, Melbourne, Victoria, Australia; 9 Murdoch Children's Research Institute, Parkville, Victoria, Australia

**Keywords:** PRISONERS, PHYSICIANS, PRIMARY CARE, HEALTH SERVICES, UTILISATION, GENERAL PRACTITIONER

## Abstract

**Objective:**

To describe the association between ex-prisoner primary care physician contact within 1 month of prison release and health service utilisation in the 6 months following release.

**Design:**

A cohort from the Passports study with a mean follow-up of 219 (±44) days postrelease. Associations were assessed using a multivariate Andersen-Gill model, controlling for a range of other factors.

**Setting:**

Face-to-face, baseline interviews were conducted in a sample of prisoners within 6 weeks of expected release from seven prisons in Queensland, Australia, from 2008 to 2010, with telephone follow-up interviews 1, 3 and 6 months postrelease.

**Participants:**

From an original population-based sample of 1325 sentenced adult (≥18 years) prisoners, 478 participants were excluded due to not being released from prison during follow-up (n=7, 0.5%), loss to follow-up (n=257, 19.4%), or lacking exposure data (n=214, 16.2%). A total of 847 (63.9%) participants were included in the analyses.

**Exposure:**

Primary care physician contact within 1 month of follow-up as a dichotomous measure.

**Main outcome measures:**

Adjusted time-to-event hazard rates for hospital, mental health, alcohol and other drug and subsequent primary care physician service utilisations assessed as multiple failure time-interval data.

**Results:**

Primary care physician contact prevalence within 1 month of follow-up was 46.5%. One-month primary care physician contact was positively associated with hospital (adjusted HR (AHR)=2.07; 95% CI 1.39 to 3.09), mental health (AHR=1.65; 95% CI 1.24 to 2.19), alcohol and other drug (AHR=1.48; 95% CI 1.15 to 1.90) and subsequent primary care physician service utilisation (AHR=1.47; 95% CI 1.26 to 1.72) over 6 months of follow-up.

**Conclusions:**

Engagement with primary care physician services soon after prison release increases health service utilisation during the critical community transition period for ex-prisoners.

**Trial registration number:**

Australian New Zealand Clinical Trials Registry (ACTRN12608000232336).

**Strengths and limitations of this study**

- This is the first study to examine the association between early primary care physician (PCP) contact and health service utilisation rates in a large prospective cohort of recently released ex-prisoners.
- Potential differences in participant characteristics between those excluded from analysis and the final study sample were empirically assessed.
- The fact that the Andersen-Gill model applied in analysis controls for both interparticipant and intraparticipant variability in event rates and the associations between early PCP contact and healthcare service utilisation remained significant after adjustment for covariates and the conservative Bonferroni adjustment further strengthens the inferences presented here.
- Cohort data used from the intervention and control arms of the Passports study were shown to influence primary care utilisation; however, these effects were controlled for in the final regression model, limiting differential bias between PCP-contact status groups.
- Other potential study limitations include a primary reliance on self-report from ex-prisoners; hospital utilisation was assessed as one broad category resulting in the inability to disaggregate emergency care from tertiary prevention contacts; a lack of data on the specific health conditions responsible for PCP-associated hospitalisations; and the contemporaneous assessment of 1-month PCP contact and other health service utilisation outcomes at 1 month follow-up.

## Introduction

Prisoners experience disproportionate health burden with prevalence estimates being over twofold higher for chronic diseases, blood-borne viruses, alcohol and other drug (AOD) disorders, and severe mental illness, compared to the community.^1^ ^2^ Previous research has demonstrated that health often improves while incarcerated,^3^ ^4^ however, it typically deteriorates, partly due to inadequate preventive measures, after prison release.^5–10^ The synergistic effect of multiple, concurrent health conditions may increase recidivism, and produce poor treatment outcomes, making management of ex-prisoners’ health especially challenging.^11^ ^12^ Not surprisingly, recent research has demonstrated a twofold increase in hospitalisation rates for ex-prisoners within 90 days of release, compared to matched community controls, with much of this increased risk attributable to ambulatory care-sensitive, and therefore preventable, conditions.^13^ ^14^

Interrupted healthcare often compounds adverse health outcomes for ex-prisoners.^15–18^ Continuity of care has been shown to increase patient appraisal of care and treatment adherence while reducing ambulatory care-related hospitalisations.^10^ ^19^ ^20^ The primary care physician (PCP), also referred to as ‘family physician’ or general practitioner in Australia, plays a pivotal role in facilitating continuity of care for vulnerable populations, such as ex-prisoners, by coordinating healthcare assessment, planning and service referral.^19^ ^21–23^ In studies of community-recruited cohorts, receiving regular, optimal primary care has been associated with increased receipt of preventive care^24^ and initial PCP contact has been demonstrated to improve subsequent healthcare seeking, access, and continuity^25^ while decreasing health inequalities.^26^ Furthermore, consistent primary care contact has been associated with decreased emergency department visits^27^ and reduced urgent care visits for those with complex comorbid health conditions in the community.^28^ Thus, facilitating PCP contact soon after prison release, establishing trust in this patient–physician relationship,^29^ and improving service continuity from prison to the community are important public health priorities for ex-prisoner healthcare. Community-based primary care can be a cost-effective way of managing healthcare for ex-prisoners.^30^

Currently, the effect of early PCP contact on subsequent health service utilisation in recently released ex-prisoners is poorly understood. Prior to inspection of the data presented here, we formed an a priori hypothesis that access to a PCP within 1 month of release from prison would be associated with increased utilisation of healthcare services in the community.

In a large sample of ex-prisoners in Queensland, Australia, the aims of the current study were to (1) estimate the prevalence of PCP contact within 1 month of release, and (2) determine the association between PCP contact in the first month postrelease and utilisation of mental health, AOD, hospital and subsequent PCP services in the first 6 months postrelease.

## Methods

### Study population

We used cohort data from the intervention and control arms of the Passports study, a randomised controlled trial of a service brokerage intervention, the design of which is described in more detail elsewhere.^31^

A baseline interview was administered to 1325 sentenced adult (≥18 years) prisoners within 6 weeks of expected release from one of seven prisons in Queensland, Australia, during the period 1 August 2008 to 31 July 2010. Three follow-up telephone interviews were conducted approximately 1, 3 and 6 months postrelease. For participants reincarcerated at the scheduled follow-up, interviews were conducted in custody. Interviews were conducted by trained researchers (N=13) with prior experience interviewing marginalised populations. All participants provided informed, written consent prior to their participation.

### Assessments

Baseline self-report measures included sociodemographic characteristics, lifetime chronic health conditions, lifetime hepatitis C exposure, social visits in the previous month while in prison, participation in transitional programmes, postrelease supervision status, and history of incarceration as a juvenile. The presence or absence of a lifetime chronic condition (ie, current and/or previous) and lifetime hepatitis C exposure (from self-reported serological or PCR test results) were assessed separately as dichotomous variables. Self-reported participation in transitional programmes, postrelease supervision orders (ie, parole and mandated treatment conditions), and incarceration history as a juvenile were assessed separately as binomial variables.

Validated screeners administered at baseline included the Patient Activation Measure (PAM)^32^ used to assess capacity for self-management of health, the Kessler Psychological Distress Scale (K10)^33^ predicting severe mental illness, and the Hayes Ability Screening Index (HASI)^34^ for the identification of possible intellectual disability (ID). The PAM score was categorised into a dichotomous severity indicator using the median value. The K10 score was collapsed dichotomously indicating low/moderate versus high/very high psychological distress.^35^ Cognitive dysfunction was identified according to the HASI screening tool, with scores <85 considered consistent with possible ID.^34^

Current central nervous system (CNS) and non-CNS medication usage was extracted from prison medical records and assessed as separate dichotomous variables; these served as proxy measures for current mental and physical health disorders, respectively.^36^ History of adult incarceration and reincarceration in the 2 years postrelease were assessed through deterministic linkage using a unique prisoner identification number from Queensland Correctional Service (QCS) records. The total number of adult prison episodes for each participant was dichotomised to indicate whether the participant had prior prison sentences. Probabilistic data linkage was conducted with the Australian National Death Index to censor deaths occurring during the follow-up period.

### Exposure—PCP contact by 1 month postrelease

The total number of self-reported PCP service utilisations within 1 month of release was dichotomised, indicating the presence or absence of PCP contact in this period.

### Study outcomes

Outcomes were self-reported utilisation of mental health, AOD, hospital, and subsequent PCP services in the community. Each service utilisation type was assessed separately at each follow-up interview. Subsequent PCP service utilisation was assessed at 3-month and 6-month follow-up. Participants reported the number of type-specific service utilisations since baseline or the previous follow-up interview, creating three distinct follow-up time intervals. The total reported service utilisations for each time interval was dichotomised to indicate the presence or absence of service utilisation at each follow-up, for each service type.

### Statistical analysis

All analyses were conducted using STATA V.13.0.^37^ All primary tests were two tailed with significance set at p<0.05. Bonferroni correction was applied to adjust for multiple testing.

Descriptive statistics were calculated for all variables. Independent samples t tests and χ^2^ analyses were performed, comparing differences between PCP-contact and no-PCP-contact groups for continuous and categorical outcomes, respectively.

The association between PCP contact at 1 month postrelease and health service utilisation over the 6-month follow-up period was estimated separately for each service type, controlling for baseline sociodemographic, behavioural, and cohort-specific characteristics (outlined below). A multivariate Andersen-Gill extension of a Cox proportional hazards model^38^ was fitted utilising robust SEs for use with multiple failure time-interval data and interval-truncation (eg, for periods of reincarceration).^39^ ^40^ The Andersen-Gill model was selected because it controls for interparticipant as well as intraparticipant variability in event rates.^38^

Time at-risk began at the initial prison release date and was censored on the last recorded follow-up interview date in the community, or at death. If the last follow-up was conducted in prison, the censor date was the preceding QCS readmission. Prison sentences that occurred within the follow-up period were truncated (ie, interval-truncation), as the participants were not ‘at-risk’ of utilising health services in the community while incarcerated. Exploratory subgroup analyses were conducted between non-recidivists and recidivists during the follow-up period, and between first-time and repeat offenders as identified at baseline.

All models were adjusted for age, gender, Indigenous status, receipt of the Passports intervention,^31^ chronic health conditions (including asthma, back problems, hypertension, high cholesterol, heart disease, diabetes and epilepsy), CNS and non-CNS medication use, social visits in prison, hepatitis C exposure status, PAM score, K10 score, ID status, participation in transitional programmes, postrelease supervision orders, history of juvenile incarceration and prior adult incarceration. Age was fitted as a quadratic covariate (ie, age squared) due to non-linearity. First-order interaction terms approaching significance (p≈0.10) were also fitted in the final model.

Missing covariate data were replaced using multiple imputation (imputed datasets: N=30) applying multivariate chained equations as described for use in the Cox model.^41^ ^42^ Overall, data were imputed for 68 participants (8.0%) with a maximum of three covariate values imputed for any one participant (table 3).

### Ethical considerations

The Passports study was approved by the University of Queensland Behavioural and Social Sciences Ethical Review Committee (Project #2007000607), QCS Research Committee, and the Queensland Health Human Research Ethics Committee (HREC/11/QHC/40), and was registered with the Australian New Zealand Clinical Trials Registry (ACTRN12608000232336).

## Results

### Participant inclusion

Seven individuals (0.5%) were excluded as they were not released from prison during the follow-up period due to parole rejection or being remanded in custody on new charges. A further 257 (19.4%) and 214 (16.2%) participants were excluded from analysis due to a complete lack of follow-up data and/or study ‘exposure’ data (ie, 1-month PCP contact), respectively. The remaining participants (N=847, 63.9%) were included in the analyses.

Compared with those excluded from analysis (N=478), this study sample was less likely to identify participants as Indigenous, significantly older, and was more likely to report social visits in prison, to have no history of juvenile incarceration, to screen negative for ID, to report having a postrelease supervisory order, to report current non-CNS medication use, to report a lifetime chronic condition, and to report no hepatitis C exposure (table 1).

**Table 1:** Passports cohort characteristics overall and by current study inclusion status

Characteristic	Study group N (%) 847 (63.9%)	Exclusion group N (%) 478 (36.1%)	Total passports cohort N (%) N=1325	Crude OR (95% CI)	p Value
Gender (N, %)					
Female	189 (22.3)	91 (19.0)	280 (21.1)		
Male	658 (77.7)	387 (82.3)	1045 (78.9)		
			1325 (100.0)	1.22 (0.92 to 1.62)	0.161*
Age (Years±SD)	34.2±11.6	31.6±9.9	33.3±11.1	2.6 (1.4 to 3.8)†	<0.001‡
Indigenous status (N, %)					
Indigenous	159 (18.8)	179 (37.5)	338 (25.5)		
Non-Indigenous	688 (81.2)	299 (62.6)	987 (74.5)		
			1325 (100.0)	0.39 (0.30 to 0.50)	<0.001*
Passports intervention (N, %)					
Yes	415 (49.0)	250 (52.3)	665 (50.2)		
No	432 (51.0)	228 (47.7)	660 (49.8)		
			1325 (100.0)	0.88 (0.70 to 1.10)	0.248*
Lifetime chronic conditions (N, %)					
Yes	581 (68.6)	302 (63.2)	883 (66.6)		
No	266 (31.4)	176 (36.8)	442 (33.4)		
			1325 (100.0)	1.27 (1.01 to 1.61)	0.045*
CNS medication use (N, %)					
Yes	244 (30.5)	128 (29.6)	372 (30.2)		
No	555 (69.5)	304 (70.4)	859 (69.8)		
			1231 (91.1)§	1.04 (0.81 to 1.35)	0.740*
Non-CNS medication use (N, %)					
Yes	244 (30.5)	104 (24.1)	348 (28.3)		
No	555 (69.5)	328 (75.9)	883 (71.7)		
			1231 (91.1)§	1.39 (1.06 to 1.81)	0.016*
Social visits in prison (N, %)					
Yes	443 (52.3)	170 (35.6)	613 (46.3)		
No	404 (47.7)	308 (64.4)	712 (53.7)		
			1325 (100.0)	1.98 (1.58 to 2.50)	<0.001*
Hep C exposure status (N, %)					
Positive	234 (27.6)	159 (33.3)	393 (29.7)		
Negative	613 (72.4)	319 (66.7)	932 (70.3)		
			1325 (100.0)	0.77 (0.60 to 0.98)	0.032*
PAM score (N, %)					
≤41	102 (12.0)	68 (14.2)	170 (12.8)		
>41	745 (88.0)	410 (85.8)	1155 (87.2)		
			1325 (100.0)	1.21 (0.87 to 1.68)	0.254*
K10 distress (N, %)					
Low/moderate	626 (74.1)	351 (73.9)	977 (74.0)		
High/very High	219 (25.9)	124 (26.1)	343 (26.0)		
			1320 (99.6)§	0.99 (0.77 to 1.28)	0.940*
HASI score <85 (N, %)					
Yes	180 (21.5)	144 (31.4)	324 (25.0)		
No	657 (78.5)	314 (68.6)	971 (75.0)		
			1295 (97.7)§	0.60 (0.46 to 0.77)	<0.001*
Transitional coordinator access (N, %)					
Yes	162 (19.1)	101 (21.2)	263 (19.9)		
No	685 (80.9)	376 (78.8)	1061 (80.1)		
			1324 (99.9)§	0.88 (0.67 to 1.16)	0.370*
Supervised after release (N, %)					
Yes	535 (63.2)	260 (54.5)	795 (60.1)		
No	311 (36.8)	217 (45.5)	528 (39.9)		
			1323 (99.9)§	1.44 (1.14 to 1.80)	0.002*
Juvenile incarceration history (N, %)					
Yes	173 (20.6)	193 (40.8)	366 (27.9)		
No	666 (79.4)	280 (59.2)	946 (72.1)		
			1312 (99.0)§	0.37 (0.29 to 0.48)	<0.001*
Adult prison sentence (N, %)					
First	340 (40.2)	104 (22.3)	444 (33.8)		
Repeat	506 (59.8)	362 (77.7)	868 (66.2)		
			1312 (99.0)§	0.43 (0.33 to 0.55)	<0.001*

*Pearson χ^2^ test.

†Mean difference (95% CI).

‡Independent t test.

§Total sums to less than 100% due to missing outcome data.

CNS, central nervous system; HASI, Hayes Ability Screening Index; Hep C, hepatitis C; K10, Kessler Psychological Distress Scale; PAM, Patient Activation Measure.

### Follow-up interview occurrence

The mean (±SD) time to each follow-up was 39 (±15), 109 (±25) and 219 (±44) days postrelease for the 1, 3 and 6-month follow-up interviews, respectively.

### One-month follow-up PCP contact

Overall, 394 participants (46.5%) reported PCP contact prior to the 1-month follow-up interview. Participant characteristics are presented overall and according to 1-month PCP contact in table 2. The majority of the cohort was male (77.7%; n=658) and the mean (±SD) age of participants was 34.2±11.6 years. Females (57.7%) were 78.3% (p<0.001) more likely than males (43.3%) to report PCP contact within 1 month. The PCP-contact group were significantly older (37.4±12.9 years) than the no-PCP-contact group (31.5±9.6 years, p<0.001) at baseline. The PCP-contact group was significantly more likely to report a lifetime chronic condition, current CNS and non-CNS medication use, and screen positive on the K10 at baseline (table 2). There was a non-significant trend for decreased 1-month PCP contact for Indigenous ex-prisoners and conversely, increased 1-month PCP contact for ex-prisoners reporting participation in transitional programmes.

**Table 2:** Cohort characteristics overall and by 1-month PCP contact status

Characteristic	PCP Contact <1 month N (%) 394 (46.5%)	No PCP contact <1 month N (%) 453 (53.5%)	All participants N (%) N=847	p Value
Gender (N, %)				
Female	109 (27.7)	80 (17.7)	189 (22.3)	
Male	285 (72.3)	373 (82.3)	658 (77.7)	
			847 (100.0)	<0.001*
Age (years±SD)	37.4±12.9	31.5±9.6	34.2±11.6	<0.001†
Age (years) (N, %)				
<25	64 (16.2)	142 (31.4)	206 (24.3)	
25–29	67 (17.0)	91 (20.1)	158 (18.7)	
30–39	128 (32.5)	137 (30.2)	265 (31.3)	
≥40	135 (34.3)	83 (18.3)	218 (25.7)	
			847 (100.0)	<0.001*
Indigenous status (N, %)				
Indigenous	63 (16.0)	96 (21.2)	159 (18.8)	
Non-indigenous	331 (84.0)	357 (78.8)	688 (81.2)	
			847 (100.0)	0.053*
Lifetime chronic conditions (N, %)				
Yes	303 (76.9)	278 (61.4)	581 (68.6)	
No	91 (23.1)	175 (38.6)	266 (31.4)	
			847 (100.0)	<0.001*
CNS medications (N, %)				
Yes	157 (42.4)	87 (20.3)	244 (30.5)	
No	213 (57.6)	342 (79.7)	555 (69.5)	
			799 (94.3)‡	<0.001*
Non-CNS medications (N, %)				
Yes	172 (46.5)	72 (16.8)	244 (30.5)	
No	198 (53.5)	357 (83.2)	555 (69.5)	
			799 (94.3)‡	<0.001*
Social visits in prison (N, %)				
Yes	204 (51.8)	239 (52.8)	443 (52.3)	
No	190 (48.2)	214 (47.2)	404 (47.7)	
			847 (100.0)	0.775*
Lifetime Hep C exposure status (N, %)				
Positive	114 (28.9)	120 (26.5)	234 (27.6)	
Negative	280 (71.1)	333 (73.5)	613 (72.4)	
			847 (100.0)	0.428*
PAM score (N, %)				
≤41	49 (12.4)	53 (11.7)	102 (12.0)	
>41	345 (87.6)	400 (88.3)	745 (88.0)	
			847 (100.0)	0.742*
K10 distress (N, %)				
Low/moderate	271 (69.0)	355 (78.5)	626 (74.1)	
High/very high	122 (31.0)	97 (21.5)	219 (25.9)	
			845 (99.8)‡	0.002*
HASI score <85 (N, %)				
Yes	89 (22.8)	91 (20.4)	180 (21.5)	
No	301 (77.2)	356 (79.6)	657 (78.5)	
			837 (98.8)‡	0.387*
Transitional programs participation (N, %)				
Yes	86 (21.8)	76 (16.8)	162 (19.1)	
No	308 (78.2)	377 (83.2)	685 (80.9)	
			847 (100.0)	0.062*
Postrelease supervision orders (N, %)				
Yes	246 (62.4)	289 (63.9)	535 (63.2)	
No	148 (37.6)	163 (36.1)	311 (36.8)	
			846 (99.9)‡	0.651*
Juvenile incarceration history (N, %)				
Yes	74 (19.0)	99 (22.1)	173 (20.6)	
No	316 (81.0)	350 (77.9)	666 (79.4)	
			839 (99.1)‡	0.272*
Adult prison sentence (N, %)				
First	164 (41.7)	176 (38.9)	340 (40.2)	
Repeat	229 (58.3)	277 (61.1)	506 (59.8)	
			846 (99.9)‡	0.394*

*Pearson χ^2^ test.

†Independent t test.

‡Total sums to less than 100% due to missing outcome data.

CNS, central nervous system; HASI, Hayes Ability Screening Index; Hep C, hepatitis C; K10, Kessler Psychological Distress Scale; PAM, Patient Activation Measure.

### One-month follow-up PCP contact and health service utilisation

**Figure 1:**
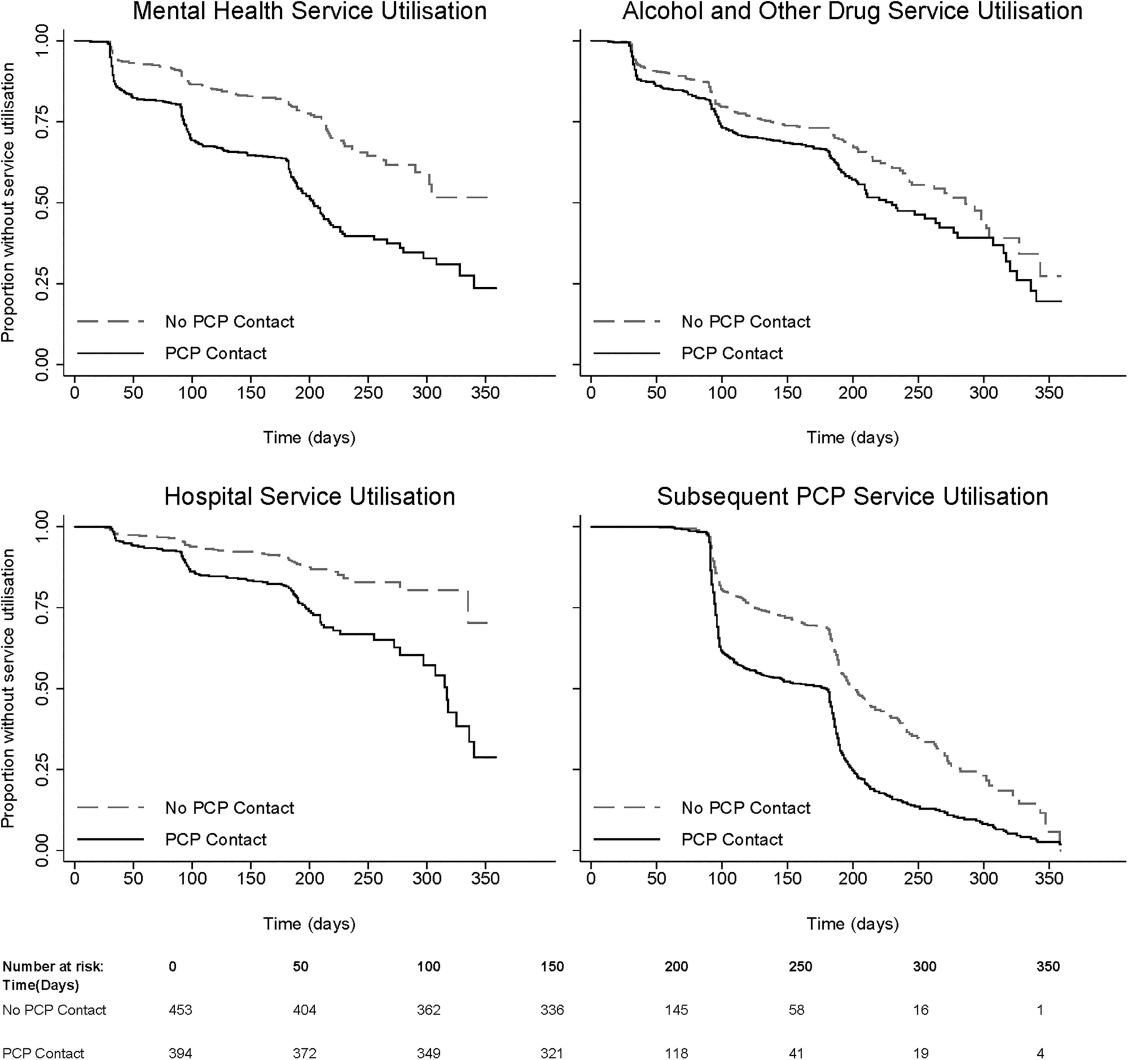
Kaplan–Meier survival curves of health service utilisation for 6 months of follow-up postrelease from prison. PCP, primary care physician.

Unadjusted Kaplan-Meier survival curves comparing type-specific service utilisation rates between the PCP-contact and no-PCP-contact groups are displayed in figure 1. Compared to the no-PCP-contact group, the PCP-contact group exhibited higher rates of utilisation of mental health, AOD, hospital and subsequent PCP services well beyond 180 days of follow-up, where the number of participants ‘at-risk’ was still substantial. Subsequent PCP service utilisation shows a delay in incidence of approximately 90 days, as assessment began at the 3-month follow-up for this outcome (figure 1).

The association between 1-month PCP contact status and health service utilisation over the follow-up period is shown in table 3. Multivariate Cox regression with imputed covariate values, adjusting for confounding and first-order interaction effects indicated that the PCP-contact group was significantly more likely to utilise health services, namely hospital (adjusted HR (AHR)=2.07; 95% CI 1.39 to 3.09), mental health (AHR=1.65; 95% CI 1.24 to 2.19), AOD (AHR=1.48; 95% CI 1.15 to 1.90), and subsequent PCP (AHR=1.47; 95% CI 1.26 to 1.72) compared with the no-PCP-contact group during follow-up (table 3). After Bonferroni correction (p<0.0125) was applied to adjust for multiple testing, all associations remained significant at p<0.05.

**Table 3:** Association between 1-month PCP contact and health service utilisation for 6 months postrelease

6-month health service utilisation	Unadjusted HR (95% CI) N=847	Adjusted HR* (95% CI) N=779	Adjusted HR with imputed values*†‡ (95% CI) N=847
Mental health services	2.34 (1.78 to 3.08)	1.68 (1.24 to 2.27)†	1.65 (1.24 to 2.19)§
Non-recidivist (N=687)	2.92 (2.13 to 4.01)	2.15 (1.55 to 2.99)‡	
Recidivist (N=160)	1.04 (0.58 to 1.86)	1.03 (0.60 to 1.79)‡	
First offenders (N=340)¶	2.71 (1.65 to 4.45)	2.10 (1.22 to 3.61)‡	
Repeat offenders (N=506)¶	2.16 (1.56 to 3.00)	1.60 (1.15 to 2.23)‡	
AOD services	1.36 (1.07 to 1.73)	1.53 (1.18 to 1.99)†	1.48 (1.15 to 1.90)§
Non-recidivist	1.51 (1.14 to 2.01)	1.48 (1.11 to 1.97)‡	
Recidivist	1.08 (0.70 to 1.65)	1.17 (0.76 to 1.81)‡	
First offenders	1.07 (0.62 to 1.85)	1.45 (0.81 to 2.62)‡	
Repeat offenders	1.46 (1.13 to 1.90)	1.36 (1.05 to 1.76)‡	
Hospital services	2.29 (1.60 to 3.29)	2.25 (1.48 to 3.43)†	2.07 (1.39 to 3.09)§
Non-recidivist	2.92 (1.86 to 4.57)	2.41 (1.46 to 3.99)‡	
Recidivist	1.28 (0.67 to 2.45)	1.03 (0.53 to 2.01)‡	
First offenders	2.65 (1.39 to 5.05)	1.97 (1.02 to 3.78)‡	
Repeat offenders	2.17 (1.40 to 3.35)	1.85 (1.12 to 3.06)‡	
Subsequent PCP services	1.88 (1.63 to 2.18)	1.52 (1.30 to 1.79)†	1.47 (1.26 to 1.72)§
Non-recidivist	2.00 (1.71 to 2.35)	1.48 (1.25 to 1.75)‡	
Recidivist	1.28 (0.86 to 1.89)	1.33 (0.86 to 2.06)‡	
First offenders	1.98 (1.58 to 2.46)	1.51 (1.19 to 1.93)‡	
Repeat offenders	1.83 (1.51 to 2.23)	1.46 (1.19 to 1.78)‡	

*Model adjusted for age, gender, Indigenous status, Passports intervention, chronic conditions, CNS and non-CNS medication use, social visits in prison, hepatitis C status, PAM score, K10 score, ID status, transitional services access, supervised release conditions, juvenile offending, prior adult incarceration.

†Model fitted with first-order interaction terms.

‡Missing covariate values (8%; N=68) replaced using multiple imputation. Covariates imputed were CNS and non-CNS medication use (N=48), HASI screening status (N=10), prior juvenile custody (N=8), K10 screening status (N=2), prior offending history (N=1), and supervised release conditions (N=1).

§Estimate significant after Bonferroni adjustment (p<0.0125) applied.

¶Subgroup analysis total sums to less than 100% of the cohort due to missing data (N=1) on prior offences.

AOD, alcohol and other drug; CNS, central nervous system; HASI, Hayes Ability Screening Index; Hep C, hepatitis C; ID, intellectual disability; K10, Kessler Psychological Distress Scale; PAM, Patient Activation Measure PCP, primary care physician.

Exploratory subgroup analysis, stratified by repeat offending, revealed that PCP contact at 1-month follow-up predicted increased rates of utilisation of hospital (AHR=2.41; 95% CI 1.46 to 3.99), mental health (AHR=2.15; 95% CI 1.55 to 2.99), AOD (AHR=1.48; 95% CI 1.11 to 1.97), and subsequent PCP services (AHR=1.48; 95% CI 1.25 to 1.75) for the non-recidivist subgroup only (table 3). Conversely, subgroup analyses showed that PCP contact status was associated with increased service utilisation for both first and repeat offenders, except that the effect on the utilisation of AOD services for first offenders did not reach statistical significance (table 3).

## Discussion

Almost half (46.5%) of the ex-prisoners in this study reported community PCP contact within 1 month of prison release. A recent Australian national survey found that 23% of prisoners had a referral or appointment with a medical practitioner on discharge, suggesting that a substantial proportion of the PCP-contact group in the current study accessed the service without prison facilitation.^2^ Importantly, the decreased 1-month PCP contact for Indigenous participants suggests that Indigenous ex-prisoners may experience more barriers to community primary care access than their non-Indigenous counterparts, which is likely to lead to concerns about the cultural appropriateness of mainstream primary care.^43^ Ex-prisoners who reported PCP consultation by 1-month postrelease were twice as likely to utilise hospital services and around 1.5 times more likely to utilise mental health, AOD and subsequent PCP services within the 6-month follow-up period, compared with ex-prisoners who reported no PCP contact. Similar findings have been observed in prison-to-community continuity of care studies focusing on HIV antiretroviral treatment adherence.^15^ ^20^

To our knowledge, this is the first study to examine the association between early PCP contact and health service utilisation in a large sample of recently released ex-prisoners. The fact that associations between early PCP contact and healthcare service utilisation remained significant after adjustment for covariates and the conservative Bonferroni adjustment further strengthens this inference. However, the results presented here must be considered in the context of some study limitations. Approximately one-third of the original study group was excluded from analysis due to a lack of exposure and/or follow-up data. However, the Passports study cohort is generally representative of all prisoners released in Queensland during the same time period^31^ and testing for informative censoring during the follow-up period indicated no bias due to loss to follow-up. Exclusion from analyses was associated with factors found to both increase and decrease 1-month PCP contact in the study analyses. Thus, it is unlikely that these exclusions would compromise the generalisability of our findings. The current study relied primarily on self-report from ex-prisoners. Nonetheless, previous research has shown that prisoner self-report of health service utilisation can be reliable.^44^ The Passports intervention, received by about half the participants, has been shown to influence PCP contact (SA Kinner, N Lennox, R Alati, *et al*, Low-intensity service brokerage increases contact with healthcare in recently released prisoners: a single-blinded, multi-site randomised controlled trial. under review), however these effects were controlled for in the final regression model, limiting possible differential bias between PCP status groups. Although we observed increased hospital utilisation among the PCP-contact group, we were unable to disaggregate this to test for differential effects on preventive healthcare (ie, tertiary prevention) and emergency care utilisation. Additionally, we were unable to determine what health conditions were responsible for PCP-associated hospitalisations. The relative contribution of these unmeasured factors is an important focus for future research. The outcome investigated was health service utilisation. Although this is suggestive of health improvement, actual health outcomes were not assessed. Lastly, 1-month PCP contact and the utilisation of other health services at 1 month were assessed contemporaneously, further complicating the process of making causal inferences. However, the two subsequent follow-up periods (3-month and 6-month) are not similarly affected.

Given that nationally, 81% of Australians access a community PCP over a 12-month period,^45^ the 1-month PCP contact prevalence observed here likely reflects the elevated healthcare needs of ex-prisoners reintegrating into the community. However, despite the high level of health need in this population, the majority of ex-prisoners in our study did not receive PCP consultation during the first month after release—a critical period for ex-prisoner healthcare planning and prevention.^16^ Although higher than in the general population, the rate of PCP contact observed here may be inadequate to meet the significant health burden experienced by recently released ex-prisoners, particularly Indigenous ex-prisoners.^15^ ^46–48^ The marginal 1-month PCP contact increase in those reporting participation in prison transitional programmes may reflect some modest benefits of transitional planning for ex-prisoner healthcare.

Compared to the no-PCP-contact group, the PCP-contact group had more chronic conditions, medication use and a greater risk of severe mental illness (ie, K10 distress). It is possible that the increased service utilisation rates we observed may reflect increased morbidity or a general propensity to access healthcare services in the PCP-contact group, such that PCP contact had no causal effect on subsequent healthcare utilisation. However, potential differential influence from these factors was controlled for in the regression model. The increased rates of utilisation of specialised care (ie, mental health and AOD), hospital and subsequent PCP services observed here suggest that early PCP contact postrelease facilitates referral to other health services, thus serving as a critical entry point into the health system for ex-prisoners reintegrating into the community. This gateway effect may be due to a combination of factors including low cost, low stigma, closer provider proximity, broader geographic distribution, easier appointment processes, pre-existing physician familiarity and reduced waiting times compared to other service types.

In participants who reoffended during follow-up, there was no significant association between PCP contact and other health service utilisation, whereas we observed significant positive associations for ex-prisoners who stayed in the community. Previous research has demonstrated the benefits of mental health, AOD and PCP service utilisation specifically related to reduced recidivism in ex-prisoner populations.^11^ ^49^ ^50^ It is possible that high frequency recidivists have many of their health needs addressed in prison, reducing their need for community healthcare. However, prior research has shown that recidivism fragments ex-prisoner community care, interrupting preventative healthcare planning and increasing barriers to access.^11^ For high-frequency reoffenders, further case management and/or more comprehensive healthcare planning and intervention is likely to be necessary.

The association between 1-month PCP contact and other health service utilisation held for both first-time and repeat offenders, except that among first-time offenders the 1-month PCP contact did not predict increased AOD service utilisation. The reasons for this are unclear. First-time offenders may be less likely to identify their substance use as problematic or conversely, they may be more likely to perceive that they can maintain prison-initiated abstinence without postrelease community health service intervention. Therefore, early PCP contact may improve continuity of care for both first-time and repeat offenders, provided they remain in the community for at least 6 months postrelease.

Prior studies have shown elevated service utilisation rates for ambulatory care-sensitive conditions in ex-prisoners^13^ and that primary care engagement reduces ex-prisoner healthcare costs.^30^ This suggests that prison resettlement programmes integrating timely, community-based PCP services may be a cost-effective way to reduce the excess public health burden attributable to ex-prisoners. There remains an urgent need to develop and rigorously evaluate such interventions, both in Australia and internationally.^51^ ^52^ Given the over-representation of Indigenous people in Australian prisons,^53^ their poorer health outcomes postrelease,^48^ and the lower 1-month PCP contact rate we observed in this group, there is a particular need for targeted, culturally-sensitive programmes to increase PCP intervention in Indigenous ex-prisoners. With Indigenous people over-represented in Australian prisons by an age-adjusted factor of 13,^53^ such interventions could play an important role in closing the health inequity gap.^54^

The current study provides previously unavailable evidence that early postrelease PCP contact increases health service utilisation in the community, especially during the period where ex-prisoners are at highest risk of poor health outcomes.^55^ Moreover, early PCP contact may aid ex-prisoners in multiple ways including creating an attainable health plan; fostering health system literacy and communication; reducing barriers and facilitating health system navigation. In effect, PCPs may be functioning as both physician and ‘case manager’.^50^ ^51^ By reducing financial barriers for ex-prisoners, contemporary healthcare reforms, such as the *Affordable Care Act* in the USA, represent a pivotal opportunity for PCPs to foster correctional healthcare partnerships, promote timely engagement and provide targeted interventions in this previously underserviced group.^56^ In this context, our findings imply that increased cooperation and integration of prison and community primary care providers aimed at maximising PCP access soon after release from prison is a public health priority.

Our results suggest that 1-month PCP service utilisation is associated with increased utilisation of mental health, AOD, hospital, and subsequent PCP services for at least 6 months after release from prison. Our findings provide new evidence that facilitating early postrelease PCP service contact may be an effective and practical way to improve ex-prisoner healthcare integration in the community. Future research should focus on understanding the factors underlying this positive association between early PCP contact and subsequent health service utilisation, in order to inform effective programmes to manage the health needs of this vulnerable population. Replication using a randomised design focused on establishing a causal relationship between early PCP contact and increased utilisation of health services by using more detailed administrative health data and evaluating the associated health outcomes, is feasible^51^ and strongly recommended.
